# Correction: PINK1 controls RTN3L-mediated ER autophagy by regulating peripheral tubule junctions

**DOI:** 10.1083/jcb.20240719305082025c

**Published:** 2025-05-19

**Authors:** Ravi Chidambaram, Kamal Kumar, Smriti Parashar, Gowsalya Ramachandran, Shuliang Chen, Susan Ferro-Novick

Vol. 223, No. 12 | https://doi.org/10.1083/jcb.202407193 | November 18, 2024

After publication, the authors discovered that an error bar had been erroneously omitted from the graph in Fig. 5 B. This graph shows that the lysosomal delivery of the ER junction marker, LNPK, was blocked in PINK1-depleted cells. An error bar has been added to the fourth column, siPINK1 + Baf. The original and corrected Fig. 5 are shown here. This error does not affect the conclusions of the study, and the figure legend remains unchanged.

This error appears in print and in PDFs downloaded before May 7, 2025. The authors apologize for any confusion this may have caused.

**Figure:**
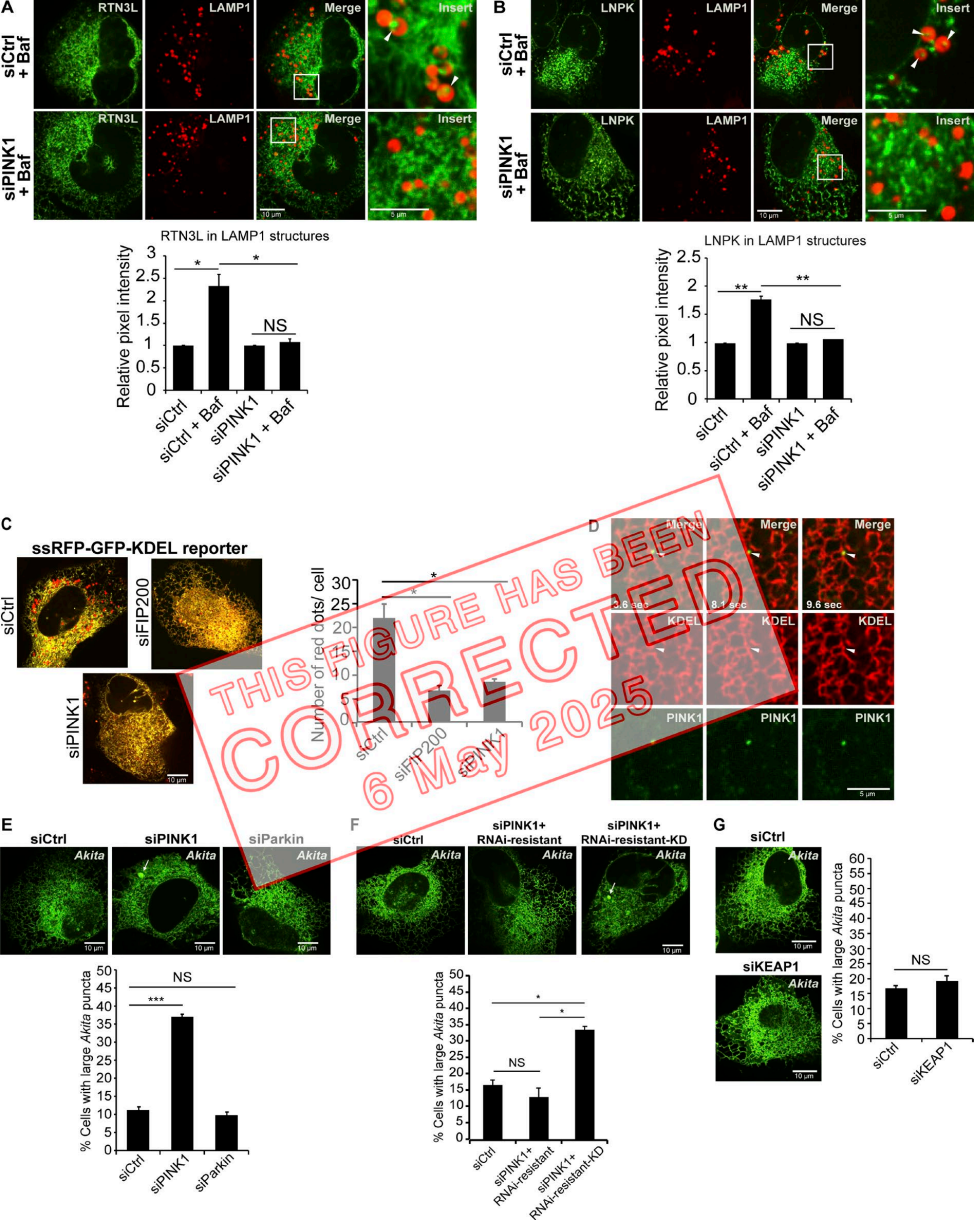


**Figure 5.**
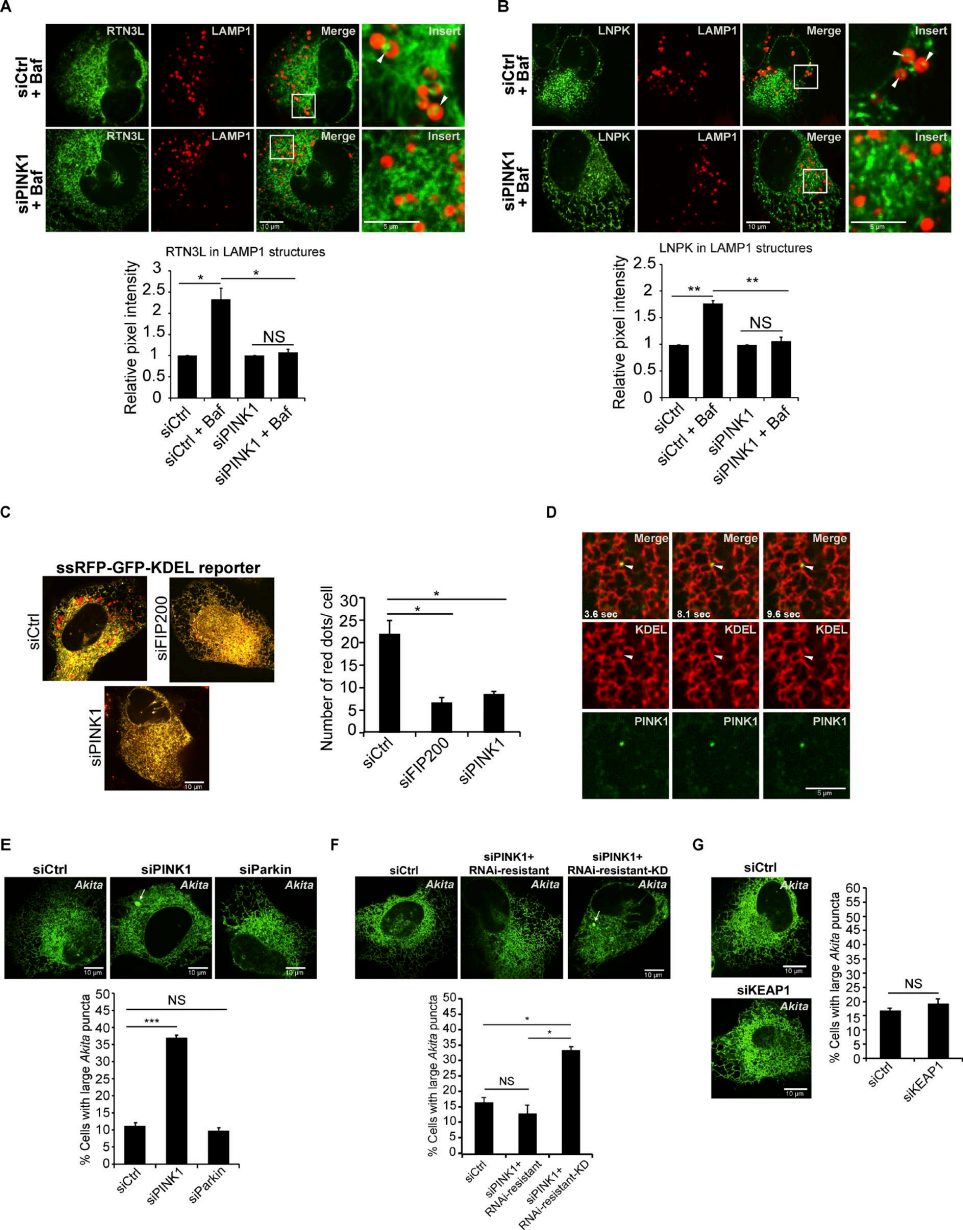
**PINK1 is needed to target tubule components for ER-phagy at three-way junctions. (A and B)** YFP-RTN3L (A) and LNPK-GFP (B) were quantitated in LAMP1-mCherry marked structures. YFP-RTN3L samples were treated with Baf for 4 h, while LNPK-GFP samples were treated for 6 h. (C) PINK1 and FIP200 are needed for the lysosomal delivery of the ER-phagy reporter ssRFP-GFP-KDEL. The number of red dots per cell are reported for the indicated samples. (D) Stills (3.6, 8.1 and 9.6 s) from Video 1 of cells transfected with mCherry-KDEL and YFP-PINK1. (E–G) Quantitation and representative images used to calculate the percent cells with large *Akita* puncta (≥0.5 µm2) in Ctrl and siRNA-treated cells. In E, the siPINK1-treated cells were transfected with mCherryRNAi-resistant PINK1 or mCherry-RNAi-resistant PINK1 kinase dead (KD). Arrows mark large *Akita* puncta. Error bars represent SEM, *n* = 3 independent experiments. The results were quantified from 58 to 69 cells in A, 47–59 cells in B, 69–85 cells in C, 99–111 cells in E, 79–105 cells in F, and 79–101 cells in G. NS: not significant (P ≥ 0.05), * (P < 0.05), **(P < 0.01), *** (P < 0.001), Student’s unpaired *t* test.

